# Paris Correspondence

**Published:** 1857-09

**Authors:** Thomas A. Means

**Affiliations:** Oxford, Georgia—now in France


ATLANTA

### Medical and Surgical Journal.

Vol. III.] SEPTEMBER, 1857. [No. 1.

### ORIGINAL COMMUNICATIONS.

### ARTICLE I.

*Parisian Hospitals—their more striking features and advantages—suggestions to American Students of Medicine who may intend to prosecute their studies, in Paris, &c.* By Thomas A. Means, M. D., Oxford, Georgia—now in France.

The general administration of the Hospitals of Paris, is so little known in America, that I design to give, for the benefit of those either interested directly or indirectly, an abridged account of this system of "Public Assistance, its doings and its advantages, believing that some good may be derived from the details furnished, especially, by those whose intention it is to visit Paris, in the pursuit of Medical knowledge.

It has been my purpose, since my arrival, (8th October last,) to communicate something upon this subject, and for the purpose of securing correct information, I have paid marked attention, both in and out of the Hospitals. Personal observation, therefore, as well as private instruction from reliable quarters, have enabled me to gain much of interest, as well as value, relative to these peculiar and most interesting benevolent institutions. Under these circumstances, therefore, I venture to submit for insertion in your Journal, the following article, trusting that the facts which it reports, may not be without value to some, who may follow me to this trans-Atlantic seat of Medical learning.

The words Hospice and Hospital are, with us, often confounded, and are regarded as one and the same, but when rightly known and considered, deserve a separate classification. An Hospice is not un-like an English Alms-house; is created and endowed by private legacies or donations, and immediately under the control and direction of the *Counsel de Surveillance*, consisting of 20 members, appointed by the National Convention, over whom the prefects of the Seine and Police preside. They decide all measures administrative, and regulate their private funds, property, &c., &c. The majority of these institutions make certain requisitions, before an entrance can be effected, for the purpose of receiving medical attention; such as absolute poverty, decrepitude and orphanage, while but little is required from the less indigent, save the payment of a sum, regulated by the comfort and position of rooms, quality of linen, bedding, &c. Thus, moderate circumstances obtain moderate accommodations. I may also add, that, persons who are not diseased, and who are anxious to curtail their expenses, are admitted upon the payment of a certain sum *par jour*, they are allowed to remain *ad libitum*, or until their funds are exhausted. Thus, those whose income is exceedingly limited, save, during a twelve month, one half of the expense, that a life in a boarding house would absolutely require. Hospitals, as Hospices, are under the control of *l'administration Général de l'Assistance, Publique;* yet they do not limit the number of their inmates, nor regard their condition, moral or financial, nor their malady. There are two grand divisions of these institutions, the former, under the head of General or Public, and the latter, special or private, while both are considered *Assistance Publique*, without any *special* difference, save the requisitions for admittance as mentioned above. Civil Hospitals, too, may be classed under the same general head, yet more directly under the control of the Minister of the Interior. There is a *Bureau Central d'Administration*, consisting of twelve Physicians and six Surgeons, who relieve each other in the discharge of special duty. This body is empowered with exclusive privileges, rejecting or admitting any patient (after examination) they may see proper, and if the disease is worthy of attention, a card of admission is given, stating the nature of the malady, occupation, age and residence of the patient. This must be presented to the Director of the Hospital, who assigns the ward confined more particularly to the disease with which he may be affected.

The principal Surgeons or Physicians attached to each Hospital, may grant admission, in urgent cases, with the signature of the Director only, and to effect this end, morning consultations, Sunday excepted, are given at 10 o'clock, after the regular ward service, to which hundreds flock for advice, many with the simplest affections possible. Children, upon Thursday of each week, are vaccinated; to the parents of whom, a sum of two or three francs is given, and those not having undergone this operation, are excluded from all the public schools, within the confines of Paris, a precaution it would be well to establish in America, and thereby prevent that public panic which throws a whole community or town into confusion, even when the simplest varioloid makes its appearance.

The number of Hospitals, exclusive of Hospices, are, at present, 26, with an aggregate of 22,482 beds; ten to twelve thousand patients of all ages and sex, are admitted annually, out of which, 4,500 are constantly under treatment, or cure: while the number of Hospices are 14—4,044 beds; patients annually admitted, 4,400; 2,000 under treatment, and the mortality 1 in 19. The yearly expenses incurred by these, amount to not less than 12,000,000 francs, or, $2,400,000. From their property, consisting of lands, houses, funds, &c., &c., comes the revenues, including the tax levied upon Theatres, and other places of amusement, together with the annual contribution of the Government. All this is in itself insufficient to supply the funds disbursed, which deficit is made up from the Public Treasury, without an additional levy upon Theatres, etc. Besides the various *Hôpitaux des maladies actuelles*, there are many others termed *Sociétés des Charités*, among which I may mention *Société Medico Philanthropique*;

*Société National de Vaccine; Société Medical du Temple; Société Medical d'Accouchement; Maisons des Sœurs l'ardes Maladies*, etc., &c. These give out-door employment to indigent families and blind persons, and gather up poor children for Christian education, Catholic or Protestant, besides teaching them a trade. They also contribute to the *Asile de la Providence*, an institution set apart for old persons, with a cost of 600 francs, each. The Minister of the Interior also pays out of the public fund 10,000 francs, annually, for its support. The most peculiar, as well as the most interesting of these Institutions, is that of the *Hospice des Infants Trouvers*, instituted by St. Vincent de Paule, between the year 30 and 34 of the 16th century; the peculiarities of which, consists in the singular, yet effective method, of receiving the foundlings. Upon the exterior of the building, there is a flanked buttress or tower: within this recess is suspended a large basket or box, called a "*tour*" attached to a rope which moves upon a pully, inside the building. A bell is also hung within, the chord of which reaches the ground without. Upon this being rung, the *tour* is turned and lowered by those within, to receive any child that may have been deposited in it, without attempting to ascertain the person or parent. There was a bill presented to the Legislature, for the suppression of this humane custom, but public opinion cried, "down with all opposition," and by an Imperial decree, issued in '54 it was withdrawn, and since then, it has been more generally adopted. It would not be amiss to state that, for two years, between the date of its origin, and the time when the bill for its suppression was introduced, its operation had been mainly discontinued. In consequence thereof, the number of infanticides, either before or after birth, were greatly augmented—in the ratio of 7 to 10, while in many localities, they were doubled and even trebled. The natural temerity and peculiar weakness of the sex, induced this crime of infanticide, from an innate dread of having their names exposed, by those ever ready to condemn their misfortunes. Thus driven to dispair, and oftentimes to madness, they put an end to the existence of the child, either before or after parturition, and thus escape public scandal.

By the means, however, of the *tours* mentioned, they are led to withhold from committing so heinous an act, and prompted by that maternal tenderness so characteristic of woman, induced, privately, to give into the hands of the charitable, the little one their misfortune had induced them to conceal.

The number of these institutions have considerably diminished since 1830, owing principally to the increase of *Maison's Publiques d'Accouchements,* to which the unfortunate females may repair without fear of public exposure, as well as receiving all necessary management and treatment during their child-bed state. Many, no doubt, possessing less of care than affection, present their offspring, as subjects of charity, rather than undergo the trouble and difficulty of rearing and supporting them. There are other establishments designed to effect like purposes, in operation in some parts of France, termed *Colonies d'Agricultures*, supported principally by the inhabitants of the district, or department in which such an institution exists. The number of foundlings received in the *Hospice des Enfants trouvers*, annually amounts to 400, and the mortality 1 in 10. The *Administration de l'Assistance Publique*, with a philanthropic spirit, which is highly commendable, have, of late, it appears, extended their sphere of usefulness, and have accomplished much towards the education of foundlings, by introducing into their system of administration certain domestic instruction. Thus, boys, when at the age of 10 or 14, are bound apprentices to some trade, where they remain a term of years, regulated by their proficiency. They are, then, set at liberty, with a small donation. Girls are likewise received—a limited number, however—who are taught those useful branches of industry best adapted to their sphere in life. Foundling girls who have served their term of years, with a character unimpeached, receive, upon marrying, a sum of 100 francs, the counsel and advice of the institution, as well as their aid in forwarding arrangements for a decent and lawful "*noce.*"

*Hospice des Orphelins* is an institution, as its name imports, set apart for the protection and care of children who have neither relations nor guardians. These are, in the French sense, considered orphans, and alone entitled to the benefits of the institution. Besides, as an asylum for orphans, they have appendêd to their code of management, a clause granting certain privileges to poor families, who, upon falling sick, are allowed to place their offspring in the institution until they are themselves cured; and if they die, their children are properly cared for, taught the elementary branches, and placed out to learn a trade until 21 years of age, when they are returned to their relatiôn or guardian who may claim them. Those under the age of 12, falling ill, are transferred to *Hôpital des Enfants Malades*, or the nearest Hospital within the arrondissement. Notwithstanding the immense number of hospitals, hospices, charitable institutions, and those confined to the treatment of special diseases, such as cutaneous, mental, &c., there is not one, nor so much as a ward, set apart for *diseases of the eye*, but the patients are scattered throughout the various "*salles*," thus placing them almost beyond the reach of him who seeks to gain information upon so important, and yet so much neglected branch of the profession. There arê four eye clinics, gotten up by private enterprise, the most important of which are those of MM. Sichel and Desmarres. The former, although younger, and less experienced, has had, during the past winter and spring, a much larger class than the latter. This justly merited popularity has excited a spirit of jealousy on the part of Desmarres, who, hitherto, occupied a position almost isolated, and was held up as the Appollo among Occulists, and the profession generally.

Sichel is a man of rather unprepossessing manners, yet mild and patient in his devotedness to the interest of his class; has no bigotry, and urges, dogmatically, no opinions; nor is he inflated with that curse of human nature—self-pride.

Desmarres, possessed with a zeal and energy peculiar to the man, has worked his way to eminence, without opposition, and is still at the head of the profession, upon the diseases of this one organ. Yet, he, like most other *specialists*, *knows too*

*much.* No credit is given his pupils for *their* knowledge, let them be ever so proficient or prompt in answering. He mocks and chuckles at the simplest blunder made, and should a correct answer be given, his vocabulary of objections would immediately supply words for an abruptly expressed, and directly opposite opinion. He takes great pride in contradicting the views of such men as Velpeau, Andral, Nelaton, Malgaigne, and others, before his class, while he quotes them with all due defference outside his clinic. This "*bonne estime de lui même*" has done him much injury, which is to be regretted, for, withal, he has thrown more light upon this one branch of the profession, perhaps, than any one who has preceded him. A student, in this department, by paying a sum of 30 francs, is entitled to a full course of two months, with the privilege of examining the patients for himself, until he is satisfied.

Most of the hospitals are on an immense scale, and some magnificent. I may mention Lariboisiére, as heading the list, completed in 1854. It contains 612 beds—ventilation most perfect—and is more in keeping, externally, with a Royal residence, than an asylum for the sick.

*St. Louis* is, perhaps, larger than any single hospital, and is devoted chiefly to cutaneous diseases, although urgent surgical cases and acute disorders are admitted. It was built by Henry IV., in 1602, and contains 853 beds; *La Charité, Hotel Dieu,* and *La Pitié,* containing, respectively, 494, 810, 624, beds, are also large and commodious. Ventilation, however, in the four last mentioned, seems to have been overlooked in their original construction and arrangement. Since '52, however, many valuable improvements and alterations have been made, both as regards proper ventilation and convenience. The utmost cleanliness prevails throughout the whole—due to the unceasing industry and vigilance of that praiseworthy sect called *Sœurs de Charité,* who devote their entire lives to the care of the sick, without the least remuneration. Many other Hospitals might be mentioned, as equally worthy of note, yet I will desist, as my article is, I fear, already too much extended. Each Hospital contains an amphitheatre—some two—

in which clinical lectures are given, gratuitously, from 9 to 10 o'clock each day, commencing in November, and ending in August. A simple ticket is all that is required for admittance, which may be obtained at the Bureau of the Hospital, upon the presentation of your Medical Diploma, or a certificate from a Professor. This requisition is not fully carried out, nor is one in fifty demanded of a student of medicine, save at *La Pitié*, and then it may be avoided.

The requisitions, before a student can receive the title of M. D., are strict and imperative.

He must be at least 18 years of age; must present the registration of his birth; a certificate from the civil authorities, of his unexceptional moral deportment—also one from his parent or guardian, announcing the step he has taken, together with his Diploma of Bachelor of Letters. He is moreover required to undergo, previous to his medical studies, a thorough examination in Greek, Latin, Rhetoric, History, Philosophy, Mathematics, and the Physical Sciences. To obtain the Diploma of Bachelor of Arts, he must be examined upon *Arithmetic*, *Geometry*, *Elementry Algebra*,—through simple equations, *Mechanics*, Elements of *Physics*, *Chemistry* and *Natural History*. After this thorough and impartial drilling, he must, before entering the medical *green room*, take what are termed subscriptions, 17 in number, for fifteen of which, he must pay 750 francs, (50 each), the 16th 35 francs; a thesis, diploma and seal, 165 francs, together with 130 francs, for the 7th, 9th, 10th, 11th and 12th inscription. The remaining eleven are not exacted; thus making a sum total of 1,100 francs, or $220.

There is an examination called *examin de fin d'année* which takes place at the expiration of the fourth year, while the *examin de doctorat* is passed, after the last payment for an inscription has been made. The studies required from a foreign student are similar to those of the secondary schools of France, and when presenting himself for examination, the elementary or secondary branches, such as Philosophy, Rhetoric, Mathematics, &c., are passed by, under the supposition that he has fully studied and prepared himself in other Universities. His

Diploma must be presented as a certificate, before an inscription, in exchange, will be granted him. The absolute and necessary expenses are the same as those of a regular French student, save the payment of 100 francs, each, for a thesis, seal, and Diploma, together with 100 francs for six "*certificates d'aptitude.*" There are three members of the Faculty appointed by the President, to conduct these examinations, the final of which, are entirely practical, and are examined at the bedside, save that of Midwifery. Two, four or six cases, as they may think proper, are selected, upon which the applicant is required to give the prognosis, diagnosis, and treatment. Those preceding upon Anatomy, are conducted over the dead body: thus, for instance, an arm, leg, or any other member is selected, who is required to dissect it in presence of the tribunal; give the particular and relative position of bones, muscles, nerves, vessels, &c. The remaining subjects, upon which he is to be examined, are drawn from an urn, so that he has no knowledge of what is to come. This is doubtless done to prevent a student from adopting any one disease, as a *speciality*, in the commencement of his medical studies. He is therefore compelled to know *well* every branch contained in the catalogue of Medical studies. His thesis must be written and printed in French, and an open defence of the views set forth therein, must be given in presence of those constituted his judges. Before taking finally the degree of M. D., he must have served the last twelve months of his course in an Hospital. Thus, briefly, are the requisitions before the title of "Doctor of Medicine" can be granted to any applicant by the French Schools. It is an ordeal, yet not so frightful as might be supposed, for many who are passed, are led by favor or preferment, answer more promptly or assume an air of confidence that wins upon the professors, while he who is less fluent, less gassy, or less prompt in answering, is turned aside, although intrinsically more deserving than his successful opponent. Thus, withal, their *réglées obligatoires*, the French Schools are not without objections. Impudence and ready speech, even in our Republic, often elevate one far beyond his actual merit —while he, whose mind as well as tongue, has to be flogged into activity, sinks infinitely lower after his *coup d'essai*, than before.

The *Faculté de Médicine* is a body of 26 professors appointed by Government, with fixed salaries, ranging from 7,500 to 10,000 francs. A Dean is also appointed for the term of five years, whose duties are not unlike those of the Dean's office in America, save in the exercise of a more absolute power: such as convoking the Faculty and suspending its action, or any Course of Lectures he may see proper.

There are also 24 "*agrégés*," chosen by competition, and are nothing more than Vice Professors. They are privileged to Lecture, conduct a chemical service, or follow the wards, in absence of the regular Professor. I give, as a note of reference, a list of the Professorships and their names attached: *Anatomy*, Denonvilliers; *Pathological Anatomy*, Chruveillier; *Physiology*, Bérard; *Surgical Pathology*, Cloquet; *General Pathology* and *Therapeutics*, Andral; *Therapeutics* and *Materia Medica*, Grisolle; *Medical Jurisprudence*, Adelon; *Hygienic Medicin*, Bouchardat; *Phamacy*, Soubeiran; *Organic Chemistry*, Wurtz; *Medical Natural Philosophy*, Gaverret; *Medical Natural History*, Moquin Tandon; *Clinical Surgery*, Velpeau, Nélaton, Jobert, and Becquerel; *Operations* and *Bandages*, Malgaigue; *Clinical Medicin*, Peorry, Bouillaud Rostan, and Trousseau; *Clinical Obstetrics*, Paul Dubois; *Obstetrics* and *Diseases* of *Females* and *Children*, Moreau; *Medical Pathology*, Duméril. This body regulates all matters pertaining to Medicine. They exercise absolute power, and are only under the control of the Government. Every position, from Interne to Professor, is determined by what are termed *concours*, or test of scholarship; and should any vacancy occur, it must be filled by him who has but succeeded in his concour, not because he may have served a term of even three score and ten years as an Interne, Externe, or Agregé, nor yet by preference, nor reputation. This system of concours is made the test then, and in some respects a rigorous one. There are certain preferences, however, allowed him who has numbered his twentieth or twenty-first year as professor in an Hospital, yet should he seek another, left vacant, either by death or resignation, he must gain his position through the *urne de scrutin*, and not by concours. Among the more prominent features in the medical schools of Paris, are the advantages in a financial point of view, an item not inconsiderable. Here a student may prosecute his medical studies for any number of years, without the cost of two francs, unless he intends graduating, and then a sum of $240 only is required. Six years is the period fixed for a full course, including two years of preliminary study, commencing with the medical alphabet and ending with a printed thesis, the smiles of the Medical Board, a Seal and Diploma. Everything is free of access. Lectures are given in the *ecole Pratique* and School of Medicine, commencing at 9 o'clock, A. M., and closing at 6 o'clock, P. M., from the first of November to the first of August. Gratuitous lectures are also given upon various medical subjects throughout the *quatrier Latin.* Dissections are not allowed, either at *ecole Pratique* or *Clamart*, the only two general dissecting rooms in the city, during the summer months; yet, courses upon Operative Surgery are permitted after a special permit has been granted the demonstrator by the *Counceil de Surveillance.*

The student, leaving America for the Paris schools, should first have obtained his diploma, (medical), and a *knowledge of the French language*, a partial knowledge *at least*; for while the *former*, acting as a passport, serves to render his position, as a physician, easy and unembarrassed, the *latter* supports his confidence and protects him from impositions. His diploma is often demanded of him. Should he fall sick and have occasion to purchase drugs of a Pharmaceutist, not one ounce can he obtain without first presenting either his diploma or a certificate from a practising physician.

The greatest drawback, however, to an English or American student, is the language, a most important consideration, and one I fear that is too much neglected. If, before reaching France, he should have gained so much as the elementary principles only, that in itself would be of inestimable value to him. By following from the day of his arrival the Clinical lectures, beside instructions and examinations, for himself, of the patients, he will have gained, before the first five months, much of value to him—information that one or two years' study in America could not give. Occasionally, a young man who has chosen Paris for a home for at least a twelve month, is impressed with the belief that he can master the language in from two to four months. To effect this, he shuts himself up, pours over his French exercises with an assiduity truly fatiguing, seldom enters an Hospital, unless for recreation, but believes, by this mechanical course of study, he will have gained the key, the oil, and the *air* of a native Frenchman, by the wane of the first moon. This assumption, provoked by self-confidence, leads him to suppose that not one expression falls from the lips of Professors or patients, that he cannot fully interpret—*mot a mot.* This is altogether a false impression, and one that instead of advancing his interest either in a medical or social point of view, throws him back in the scale of advancement, besides robbing him of his most valuable moments—moments that might have been profitably spent in a free intercourse with the masses. Some *few* there are, I will admit, who gain a sufficient knowledge within the space of three months to *understand* with tolerable facility, but who, when attempting to *speak*, make most hideous blunders, more especially in the construction and disposition of the partitive articles and prepositions. Let five months pass, and he will tell you he knows less of the language he supposed he knew than in the first two weeks or ten days. I do not intend this to discourage others from coming here, but merely to put them upon the *qui vive*, and above all to urge the necessity of studying the language at home. I would deferentially advise, also, that a student should, by all means, bring his English works, at least upon Surgery, Physiology, Obstetrics and Practice of Medicine, for he will find them invaluable aids, especially during the early part of his medical course. After he has learned to read with facility, he can consult those of French authors. While noticing the difficulties that one must necessarily encounter, I must not omit that of *living*, taken in an American sense. Everything from the most ordinary *necessities* to the smallest luxuries, are exorbitantly high, and in scarcely one article is there an exception to the remark. You will be told, doubtless, the contrary, but you should prepare yourselves with a *surplus* for emergencies, let the dream of ease, comfort, good-living, &c., be what it may. *Books*, (unbound,) *gloves*, and *wine* are about the only cheap articles of merchandise. Notwithstanding these inconveniences, a life among the Hospitals is a plesant though laborious one.

Withal, France stands *first* in Medicine and Surgery; its Hospital system bette rregulated; its requisitions for graduation more severe, and its board of instruction, coupled with an insatiable thirst for medical investigation, places her far beyond any other country. England, I fear, has some national characteristics which must ever act as drawbacks upon her success in attempting to rival her sister across the channel. She has, it is true, turned out many distinguished men in the profession, and has done much towards the advancement of medicine, but by whom? Men of poverty and low birth? Rarely ever but by men claiming to be the descendants of Lords or Duchesses; thus, if one drop of royal *sang* can be detected in his genealogy, he is at once favored and promoted. Lineage, not worth or merit, are the requisites for promotion or advancement. There is one feature in the French schools I would wish to see adopted in those of America; that is, the absence of all applause, filth, tobacco juice, disorder, and an excess of wine. The most profound silence in the wards and amphitheatres is observed, almost to a fault. What an opposite state of things exists in a majority of our schools, North and South. Repeated applause is tolerated, if not approved. A spirit of restlessness too often manifests itself, with a censurable inattention to the valuable instructions of the worthy professor, who is sometimes compelled to lecture while a portion of his class are either taking a private game at cards, satisfying the inner man with a mess of pea-nuts, and strewing the hulls about the floor, or it may be, indulging in a quiet slumber; and yet, we would not turn *censor morum*, and criticise the habits of our compeers in the honored land of our common nativity. No ! no ! while we would gladlysee American students surpassed by none on earth, and would gladly hail any rational improvement, we are too young to censure, and have no taste for the work. Facts and opinions then, have been frankly, perhaps too frankly, given, but should the hasty and unsystematised suggestions here furnished, aid any of my beloved young countrymen in their attempts to prosecute their medical studies abroad, and especially in the great French capital, I shall be amply compensated for supplying these details.

